# Th1 and Th17 Cells and Associated Cytokines Discriminate among Clinically Isolated Syndrome and Multiple Sclerosis Phenotypes

**DOI:** 10.3389/fimmu.2017.00753

**Published:** 2017-06-30

**Authors:** Gabriel Arellano, Eric Acuña, Lilian I. Reyes, Payton A. Ottum, Patrizia De Sarno, Luis Villarroel, Ethel Ciampi, Reinaldo Uribe-San Martín, Claudia Cárcamo, Rodrigo Naves

**Affiliations:** ^1^School of Medicine, Institute of Biomedical Sciences (ICBM), Universidad de Chile, Santiago, Chile; ^2^Faculty of Science, Universidad San Sebastián, Santiago, Chile; ^3^Department of Neurology, University of Alabama at Birmingham, Birmingham, AL, United States; ^4^Department of Public Health, Pontificia Universidad Católica de Chile, Santiago, Chile; ^5^Department of Neurology, Pontificia Universidad Católica de Chile, Santiago, Chile; ^6^Neurology Service, Hospital Sotero del Río, Santiago, Chile

**Keywords:** multiple sclerosis, cytokines, Th1 cells, Th17 cells, biomarker, clinical isolated syndrome, relapsing–remitting multiple sclerosis, progressive multiple sclerosis

## Abstract

Multiple sclerosis (MS) is a chronic, inflammatory, and demyelinating disease of the central nervous system. It is a heterogeneous pathology that can follow different clinical courses, and the mechanisms that underlie the progression of the immune response across MS subtypes remain incompletely understood. Here, we aimed to determine differences in the immunological status among different MS clinical subtypes. Blood samples from untreated patients diagnosed with clinically isolated syndrome (CIS) (*n* = 21), different clinical forms of MS (*n* = 62) [relapsing–remitting (RRMS), secondary progressive, and primary progressive], and healthy controls (HCs) (*n* = 17) were tested for plasma levels of interferon (IFN)-γ, IL-10, TGF-β, IL-17A, and IL-17F by immunoanalysis. Th1 and Th17 lymphocyte frequencies were determined by flow cytometry. Our results showed that IFN-γ levels and the IFN-γ/IL-10 ratio were higher in CIS patients than in RRMS patients and HC. Th1 cell frequencies were higher in CIS and RRMS than in progressive MS, and RRMS had a higher Th17 frequency than CIS. The Th1/Th17 cell ratio was skewed toward Th1 in CIS compared to MS phenotypes and HC. Receiver operating characteristic statistical analysis determined that IFN-γ, the IFN-γ/IL-10 ratio, Th1 cell frequency, and the Th1/Th17 cell ratio discriminated among CIS and MS subtypes. A subanalysis among patients expressing high IL-17F levels showed that IL-17F and the IFN-γ/IL-17F ratio discriminated between disease subtypes. Overall, our data showed that CIS and MS phenotypes displayed distinct Th1- and Th17-related cytokines and cell profiles and that these immune parameters discriminated between clinical forms. Upon validation, these parameters might be useful as biomarkers to predict disease progression.

## Introduction

Multiple sclerosis (MS) is an inflammatory and demyelinating disorder that affects the central nervous system. It is characterized by different clinical manifestations and an unpredictable clinical course. The disease frequently begins with a first episode of neurological disturbance known as clinically isolated syndrome (CIS). Then, in most cases, a relapsing–remitting disease (RRMS) develops. Within 20 years, the majority of RRMS patients will convert to secondary progressive MS (SPMS), characterized by a progressive accumulation of neurological damage with or without relapses (1). Only 10–15% of patients develop primary progressive MS (PPMS) from onset, which begins with a progressive and chronic disease course without relapses.

Clinical, epidemiological, immunological, histopathological, and imaging evidence suggest that relapse- and progressive-onset diseases are led by distinct effector pathways (2–5). Such observations have led to the notion that RRMS is driven by inflammatory processes, whereas the accumulation of disability in progressive diseases is promoted by neurodegeneration independent of inflammation. This conclusion is supported by the lack of effectiveness of current anti-inflammatory and disease-modifying therapies (DMTs), such as treatment with interferon (IFN)-β, after transition to SPMS (6, 7), and PPMS (8). In contrast, several studies have demonstrated that inflammation is still relevant and closely associated with axonal injury in progressive MS (9–13). Overall, there is limited and controversial information characterizing the immune status among different MS phenotypes (14–17) and the contribution of the immune system in disease progression remains incompletely understood. Even more, no biomarker discriminating between MS clinical forms has been validated until now.

Cytokines produced by different subtypes of T helper (Th) cells are critical components of the inflammatory process and active players in MS development. While Th1 (IFN-γ)- and Th17 (IL-17)-related cytokines have been involved in disease onset and progression, regulatory cytokines such as IL-10 and TGF-β have been associated with anti-inflammatory effects and the improvement of symptoms (18, 19). Several studies have demonstrated the heterogeneity of cytokine and chemokine levels among MS phenotypes (15–17) and their relationship with responsiveness to IFN-β treatment (15, 20, 21).

Given that MS is a complex heterogeneous disease, we have hypothesized that the immune response may dynamically change across disease course. In this cross-sectional study, we determined plasma levels of Th1-, Th2-, Th3-, and Th17-associated cytokines and Th1 and Th17 cell frequencies in untreated patients with CIS and different clinical forms of MS. We found that these clinical subtypes exhibit an altered and distinct immune response that would evolve from a Th1 into a Th17 phenotype as disease progresses from CIS to RRMS or to PPMS. More importantly, we determined, for the first time, that IFN-γ and the ratio between some cytokines can represent a biomarker to discriminate between MS phenotypes. Reproduction of our findings on a larger scale could validate the usefulness of these parameters as biomarkers for differential MS diagnosis and disease progression.

## Materials and Methods

### Patients

Eighty-three patients attending the MS Program at the Department of Neurology, Pontificia Universidad Católica de Chile, were included in the study. Twenty-one patients were diagnosed with CIS and later diagnosed as RRMS, and 62 were diagnosed with clinically definite MS (34 RRMS, 11 SPMS, and 17 PPMS). Seventeen healthy volunteer individuals were used as a control group (HC). MS diagnosis and clinical course of disease were defined according to the revised McDonald criteria (22) and Lublin and Reingold (23), respectively. Clinical examination and magnetic resonance imaging of brain and spinal cord were performed in all patients before inclusion in the study. Disability was assessed by Kurtzke’s Expanded Disability Status Scale (24). Patients with symptoms of acute systemic inflammation and inflammatory neurological diseases other than MS and patients receiving immunomodulating medications within the previous 3 months were excluded. One patient had received IFN-β treatment for a few months within the past year before the sample was taken. Blood samples were collected before beginning any immunomodulatory therapy, and patients who were at least 2 weeks postrelapse were not undergoing corticosteroid treatment. The study was approved by the Ethics Committee of the Catholic University’s Clinical Hospital, and all patients signed a written informed consent in accordance with the Declaration of Helsinki.

### Plasma Collection and Cell Activation

Venous blood was collected in Vacutainer tubes (BD Biosciences) containing EDTA. Plasma samples were obtained, aliquoted, and stored at −80°C until assessed. Simultaneously, from the same patients, peripheral blood mononuclear cells (PBMC) were isolated from heparinized blood by Ficoll-Hypaque density gradient centrifugation (GE Healthcare). A few patients were unable to donate blood for both plasma and PBMC analyses, and in those cases, only plasma was collected. Cells were immediately cultured and either untreated (nonactivated control) or treated with 1 µg/ml of anti-CD3/CD28 mAB (CD3/CD28) (eBioscience) for 72 h.

### Cytokine Measurement

Plasma sample aliquots were thawed and used only once. Levels of IFN-γ, IL-10, TGF-β, IL-17A, and IL-17F were measured by ELISA using commercial kits from eBioscience. IL-17A and IL-17F were also assessed using an ELISA kit from R&D Systems, Inc. The data depicted in the figures and used for analysis were obtained from the eBioscience kit for IL-17F and the R&D Systems, Inc., kit for IL-17A. All samples were assayed in duplicate.

### Flow Cytometry

Four hours before the completion of PBMC activation, cells were restimulated with 5 µg/ml of Brefeldin A (BFA), 50 ng/ml of phorbol 12-myristate 13-acetate (PMA), and 500 ng/ml of ionomycin (all from Sigma-Aldrich) and were added to the cell cultures in order to restimulate and retain intracellular cytokine expression. Nonactivated cells were stimulated only with BFA (nonactivated control) to determine the basal level of intracelullar cytokines. The cells were next labeled for CD4, fixed and permeabilized with Cytofix/Cytoperm (BD Biosciences), and then stained for IFN-γ and IL-17A. Cells were analyzed in an FACSVerse flow cytometer (BD Biosciences) using the FCS Express 4 Research Plus Edition software (*De Novo* Software).

### Statistical Analysis

Differences between groups were tested using the nonparametric Kruskal–Wallis rank sum test, followed by Mann–Whitney *U* test, with Dunn’s correction for multiple comparisons, if the former indicated significant differences. The Spearman rank correlation test was used to ascertain the associations between immune and clinical parameters. Receiver operating characteristic (ROC) curves were used to examine the predictive discriminating values. The Youden index was calculated to determine the cutoff value, which maximizes discriminating accuracy. *p*-Values <0.05 were considered statistically significant. Data were analyzed using the GraphPad Prism v. 5.03 (San Diego, CA, USA).

## Results

### Altered Plasma Cytokine Levels in CIS and MS Phenotypes

**Figure 1:**
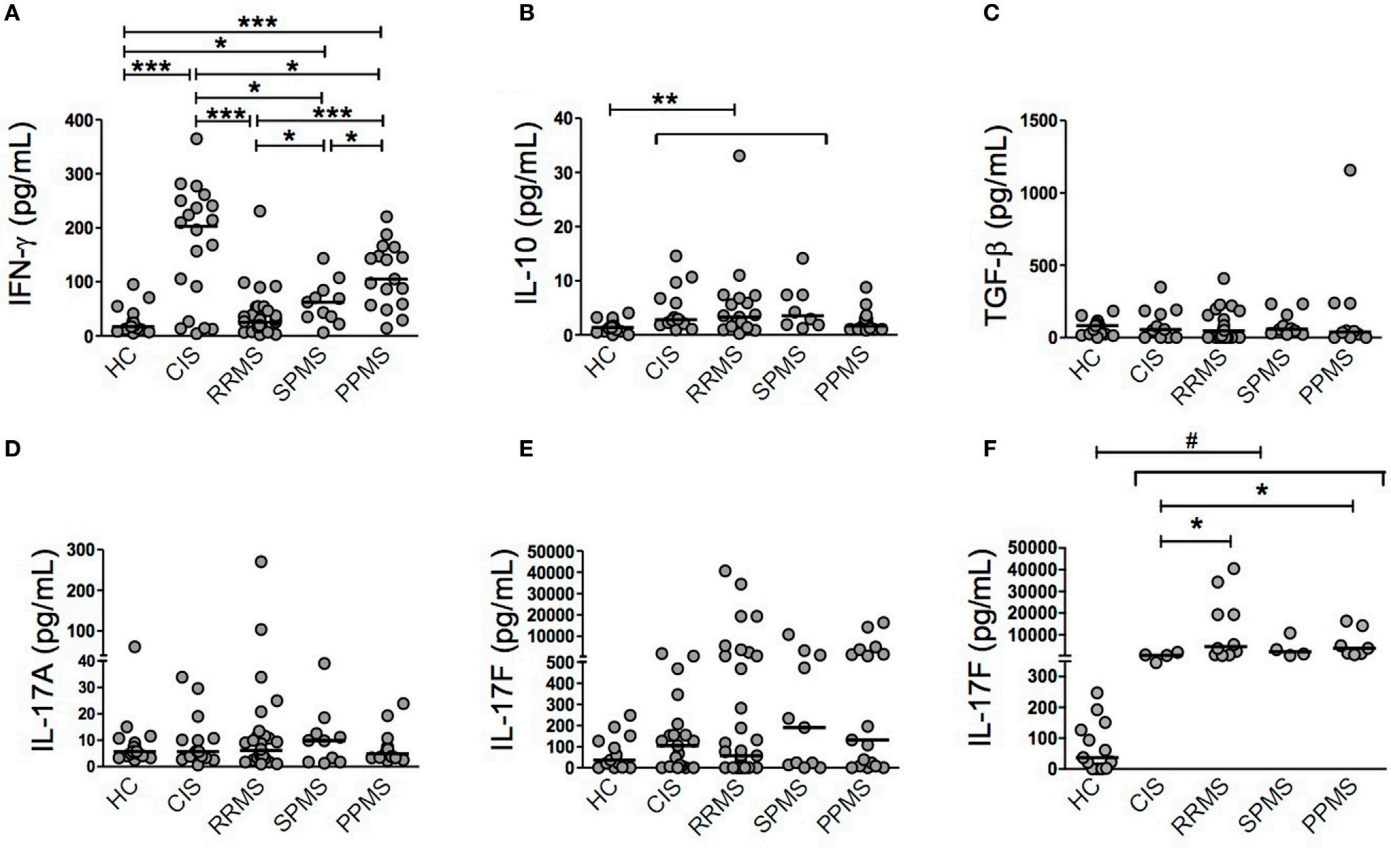
Individual cytokine production in patients with clinically isolated syndrome (CIS) and different multiple sclerosis (MS) phenotypes. Secretion of cytokines was determined in plasma samples from CIS, relapsing–remitting MS (RRMS), secondary progressive MS (SPMS), primary progressive MS (PPMS) patients, and healthy control (HC) individuals by ELISA. Some patients were found below the detection limit in all patient groups. **(A)** Interferon (IFN)-γ (HC, *n* = 17; CIS, *n* = 20; RRMS, *n* = 34; SPMS, *n* = 11; PPMS, *n* = 17), **(B)** IL-10 (HC, *n* = 17; CIS, *n* = 14; RRMS, *n* = 19; SPMS, *n* = 8; PPMS, *n* = 14), **(C)** TGF-β (HC, *n* = 15; CIS, *n* = 11; RRMS, *n* = 21; SPMS, *n* = 10; PPMS, *n* = 10), **(D)** IL-17A (HC, *n* = 17; CIS, *n* = 17; RRMS, *n* = 28; SPMS, *n* = 10; PPMS, *n* = 17), **(E)** IL-17F (HC, *n* = 13; CIS, *n* = 21; RRMS, *n* = 34; SPMS, *n* = 11; PPMS, *n* = 17), and **(F)** a subgroup of CIS and MS patients with IL-17F levels above 250 pg/ml was analyzed separately (HC, *n* = 13; CIS, *n* = 4; RRMS, *n* = 10; SPMS, *n* = 4; PPMS, *n* = 7). Horizontal line represents the median of each patient group and HC. ^#^IL-17F levels of HC were significantly lower than each patient group: *p* = 0.0038 (HC vs CIS and SPMS), *p* < 0.0001 (HC vs RRMS), and *p* = 0.0004 (HC vs PPMS). *p < 0.05, **p < 0.01, and ***p < 0.001.

First of all, we were interested in determining the immune status among CIS patients and different MS phenotypes. To avoid the influence of therapeutic interventions on real immune system disturbances, untreated patients were analyzed. The demographic and clinical features of patients and HC are summarized in Table 1. Importantly, there was no statistically significant difference between the times since last relapse between CIS and RRMS groups (Table 1). Plasma samples were tested for IFN-γ, IL-10, TGF-β, IL-17A, and IL-17F. The IFN-γ level clearly and sequentially distinguished among patient groups (Figure 1A). CIS patients exhibited significantly higher IFN-γ production than PPMS (*p* = 0.0349), SPMS (*p* = 0.0219), and RRMS (*p* = 0.0004) patients and HC (*p* = 0.0007). Of note, these results did not change after excluding CIS and RRMS patients who had a recent relapse (within 1 month of blood draw) from the analysis. PPMS patients had significantly increased IFN-γ secretion compared to SPMS (*p* = 0.0305) and RRMS (*p* < 0.0001) patients and HC (*p* = 0.0001). SPMS, in turn, showed significantly higher levels of IFN-γ than RRMS (*p* = 0.0201) and HC (*p* = 0.0127). Similar IFN-γ production was found between RRMS and HC. IL-10 and TGF-β levels were often found below the detection limit in all patient groups (Figures 1B,C). IL-10 levels were similar among CIS, RRMS, and SPMS, and they were significantly higher than PPMS (*p* = 0.0186, *p* = 0.0149, *p* = 0.0247, respectively) and HC (*p* = 0.0014, *p* = 0.0006, *p* = 0.0080, respectively; Figure 1B). TGF-β levels were similar among MS phenotypes and HC (Figure 1C). Consistent with previous studies (25, 26), IL-17A expression was often found below the detection limit in all patient groups (Figure 1D), despite use of two different commercial ELISA assays. Although there were detectable levels of IL-17F, the differences were not significant between groups (Figure 1E). However, a subgroup of CIS and MS patients producing over 250 pg/ml of IL-17F had significantly higher levels than HC (CIS, *p* = 0.0038; RRMS, *p* < 0.0001; SPMS, *p* = 0.0038; PPMS, *p* = 0.0004; Figure 1F). Within this subgroup, RRMS and PPMS patients exhibited significantly higher IL-17F levels than CIS patients (*p* = 0.0337 and *p* = 0.0424, respectively; Figure 1F).

**Table 1:** Demographic and clinical characteristics of MS patients and HC.

Characteristics	CIS (*n* = 21)	RRMS (*n* = 34)	SPMS (*n* = 11)	PPMS (*n* = 17)	HC (*n* = 17)
Male/female	7/14	10/24	3/8	7/10	7/10
Age (years), mean (SD)	31.7 (8.15)	32.8 (9.9)	42.2 (10.1)	56.4 (8.82)	33.6 (12.4)
Disease duration (years), mean (SD)	0.7 (0.98)	4.7 (4.2)	11.7 (5.7)	15.2 (10.6)	…
EDSS, mean (SD)	1 (0.95)	1 (0.93)	6 (1.53)	6 (2.22)	…
Time since last relapse (months), mean (SD)	8.3 (13.04)	4.2 (6.75)	…	…	…

*MS, multiple sclerosis; CIS, clinical isolated syndrome; RRMS, relapsing–remitting multiple sclerosis; SPMS, secondary progressive multiple sclerosis; PPMS, primary progressive multiple sclerosis; HC, healthy controls; EDSS, Expanded Disability Status Scale*.

### Distinct Plasma Cytokine Ratios in CIS and MS Phenotypes

**Figure 2:**
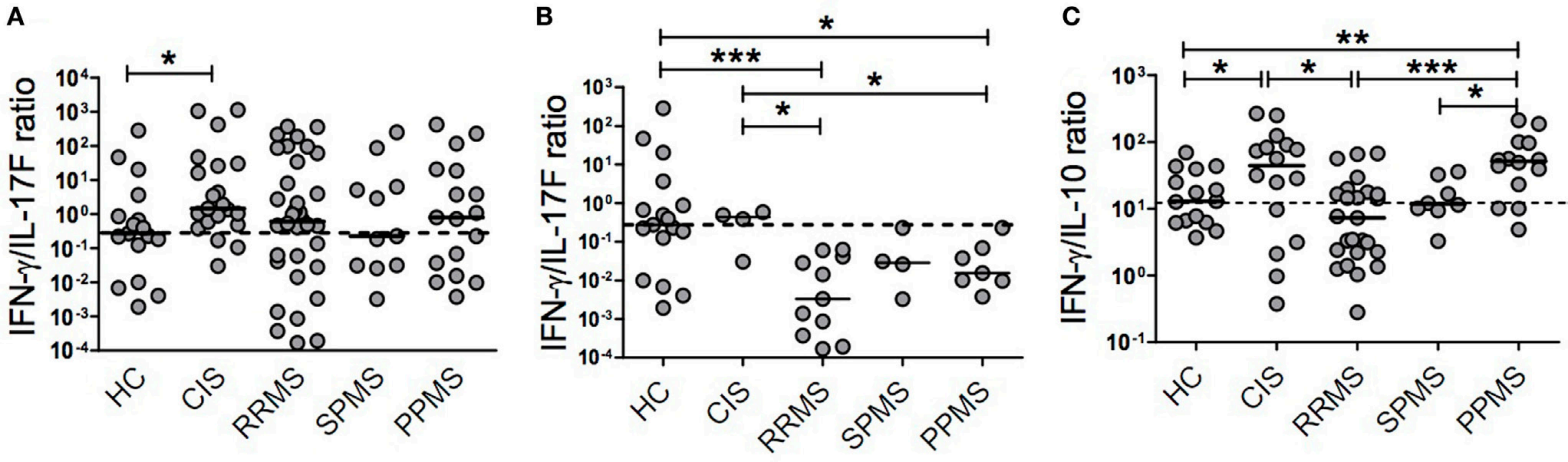
Plasma cytokine ratios in patients with clinically isolated syndrome (CIS) and different multiple sclerosis (MS) phenotypes. The relative production of cytokines was calculated in patients with CIS (*n* = 21), relapsing–remitting MS (RRMS, *n* = 34), secondary progressive MS (SPMS, *n* = 11), primary progressive MS (PPMS, *n* = 17), and healthy control (HC, *n* = 17) individuals. **(A)** Interferon (IFN)-γ/IL-17F ratio, **(B)** IFN-γ/IL-17F ratio in a subgroup of CIS and MS patients with high IL-17F levels, and **(C)** IFN-γ/IL-10 ratio. Dotted horizontal line represents the median of the respective cytokine ratio in HC. The *y*-axis in each graph is represented by logarithmic scale (*p < 0.05, **p < 0.01, and ***p < 0.001).

Given the distinctive relationship between cytokines and CIS and MS subtypes, we hypothesized that relative cytokine levels could be more informative than absolute levels in reflecting the evolution of the immune response across MS. The corresponding reanalysis revealed that patients with different MS phenotypes displayed distinct IFN-γ/IL-17F and IFN-γ/IL-10 ratios (Figures 2A–C). CIS patients had a significantly higher IFN-γ/IL-17F ratio compared to HC (*p* = 0.0118). RRMS, SPMS, and PPMS patients exhibited slightly lower ratios of IFN-γ/IL-17F than CIS patients (Figure 2A). Analysis of the subgroup of patients expressing high IL-17F levels revealed significantly lower IFN-γ/IL-17F ratios in RRMS and PPMS than in CIS (*p* = 0.0157 and *p* = 0.0424, respectively) and HC (*p* = 0.0007 and *p* = 0.0421, respectively; Figure 2B). Interestingly, the comparison of median values of the IFN-γ/IL-17F ratio among CIS and different MS stages (Figures 2A,B) suggests that MS might skew from a Th1 phenotype toward a Th17 as disease progresses from CIS (all group median = 290.8, subgroup median = 54.6) into RRMS (all group median = 120.7, subgroup median = 0.7) or PPMS (all group median = 160.8, subgroup median = 3.1). The IFN-γ/IL-10 ratio was significantly higher in CIS patients than in RRMS patients and HC (*p* = 0.0156 and *p* = 0.0491, respectively) and in PPMS patients than in RRMS and SPMS patients and HC (*p* = 0.0006, *p* = 0.0185, and *p* = 0.0083, respectively; Figure 2C).

We did not find an association between levels of cytokines or their ratios and demographic or clinical parameters among any patient groups.

### Distinct Th1 and Th17 Immune Response in CIS and MS Phenotypes

**Figure 3:**
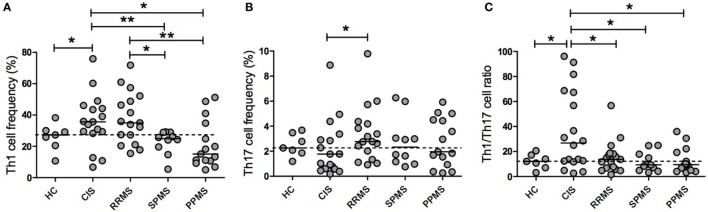
Th1 and Th17 cell frequencies and their ratios in patients with clinically isolated syndrome (CIS) and multiple sclerosis (MS) phenotypes. Purified peripheral blood mononuclear cells from CIS and MS patients and healthy control (HC) individuals were *ex vivo* stimulated with 1 µg/ml anti-CD3/CD28 antibodies (CD3/CD28) for 72 h. Intracellular expression of interferon (IFN)-γ and IL-17A was determined by flow cytometry. The results are shown as the median of the percentage of CD4^+^ T lymphocytes producing **(A)** IFN-γ (Th1) or **(B)** IL-17A (Th17). **(C)** The Th1/Th17 cell ratio was expressed as the ratio of the percentage of CD4^+^ T lymphocytes producing IFN-γ to those of IL-17A of each patient and HC. Dotted horizontal line represents the median of the Th1/Th17 ratio of HC. CIS (*n* = 17), relapsing–remitting MS (RRMS, *n* = 17), secondary progressive MS (SPMS, *n* = 10), primary progressive MS (PPMS, *n* = 15), and HCs (*n* = 7). In a few patients, it was not possible to obtain sufficient blood sample to analyze both plasma cytokine levels and cell frequencies, and therefore, they were not included in the Th1 and Th17 analyses (*p < 0.05 and **p < 0.01).

Given that many cell types (i.e., CD8^+^ and CD4^+^ T cells and NK cells) produce IFN-γ and IL-17 (27) and that Th1 and Th17 cells are considered key players in the immunopathogenesis of MS, we analyzed the frequency of Th1 and Th17 cells in a subset of MS patients after *ex vivo* CD3/CD28 stimulation. A representative flow cytometry gating strategy is illustrated in Figure S1 of the Supplementary Material. Figure 3A shows that CIS patients exhibited a significantly higher frequency of CD4^+^IFN-γ^+^ T cells than HC (*p* = 0.0328) and SPMS (*p* = 0.0062) and PPMS (*p* = 0.0314) patients. Interestingly, RRMS patients had a significantly higher Th1 cell frequency than SPMS (*p* = 0.0149) and PPMS (*p* = 0.0041) patients. In contrast, the frequency of Th17 cells was lower in CIS patients than in RRMS patients (*p* = 0.0496; Figure 3B). Remarkably, CIS patients had a significantly higher Th1/Th17 ratio than HC and RRMS, SPMS, and PPMS patients (*p* = 0.0480, *p* = 0.0311, *p* = 0.0293, and *p* = 0.0112, respectively; Figure 3C). Therefore, the Th1/Th17 cell ratio along with the IFN-γ/IL-17F cytokine ratio analysis suggests a progression from a Th1 phenotype toward a Th17 phenotype as disease evolves from CIS to RR or PP subtypes.

### Discriminating Value of Plasma IFN-γ and the IFN-γ/IL-10 Ratio for CIS and MS Phenotypes

To assess whether evaluated cytokines and their ratios discriminate between CIS, MS, and different MS phenotypes, we performed ROC analysis (Table 2). Levels of IFN-γ significantly discriminated patients with CIS from MS, RRMS, and HC [area under curve (AUC) = 0.76, 0.80, and 0.84, respectively], RRMS from SPMS (AUC = 0.74), and PPMS from HC (AUC = 0.91). The IFN-γ/IL-10 ratio significantly differentiated patients with CIS from RRMS (AUC = 0.72) and RRMS and PPMS from HC (AUC = 0.76 and 0.71, respectively). The IFN-γ/IL-17F ratio was borderline significant to discriminate CIS from HC (*p* = 0.05629). Furthermore, among the subgroup of patients with high IL-17F levels, this cytokine discriminated HC from CIS (AUC = 1.0) and from MS both combining MS phenotypes and considering them separately [AUC = 1.0 (MS), 1.0 (RRMS), 1.0 (SPMS), and 1.0 (PPMS)]. In the same subgroup, both the IL-17F level and the IFN-γ/IL-17F ratio discriminated CIS from MS, RRMS, and PPMS (IL-17F: AUC = 0.87, 0.89, and 0.89, respectively; IFN-γ/IL-17F: AUC = 0.91, 0.93, and 0.89, respectively). That subgroup’s IFN-γ/IL-17F ratio also distinguished between HC and MS, RRMS, or PPMS (AUC = 0.83, 0.89, or 0.77, respectively).

**Table 2:** Discriminating value of cytokines and cytokine ratios for CIS, MS, and MS stages.

Biomarker	Stages	AUC	Sensitivity (%)	Specificity (%)	Cut-off (pg/ml)	95% CI	*p*-Value
IFN-γ	CIS vs HC	0.84	76.2	93.3	81.2	0.71–0.90	0.00056
IFN-γ	CIS vs MS^a^	0.76	91.2	66.7	152.2	0.60–0.90	0.00047
IFN-γ	CIS vs RRMS	0.80	93.1	76.2	90.7	0.65–0.90	0.00039
IFN-γ	RRMS vs SPMS	0.74	81.8	65.5	140	0.55–0.90	0.023
IFN-γ	PPMS vs HC	0.91	82.4	86.7	56	0.82–1.00	<0.0001
IFN-γ/IL-10	CIS vs RRMS	0.72	90	69	22.4	0.53–0.90	0.02194
IFN-γ/IL-10	RRMS vs HC	0.71	80	54	17	0.53–0.80	0.04278
IFN-γ/IL-10	PPMS vs HC	0.76	71.4	77	39.4	0.57–0.90	0.01989
IFN-γ/IL-17F	CIS vs HC	0.69	73.3	71.4	0.89	0.51–0.80	0.05629
**Subgroup of patients expressing high levels of IL-17F**							
IL-17F	MS^a^ vs HC	1.0	100	100	357.5	1.0–1.0	<0.0001
IL-17F	CIS vs HC	1.0	100	100	296.4	1.0–1.0	0.00326
IL-17F	CIS vs MS^a^	0.87	81.8	65.5	2,167	0.69–1.0	0.02161
IL-17F	CIS vs RRMS	0.89	70	100	2,167	0.71–1.0	0.02842
IL-17F	CIS vs PPMS	0.89	100	75	773.1	0.53–0.90	0.03769
IL-17F	RRMS vs HC	1.0	100	100	357.5	1.0–1.0	<0.0001
IL-17F	SPMS vs HC	1.0	100	100	359.6	1.0–1.0	0.00326
IL-17F	PPMS vs HC	1.0	100	100	535.1	1.0–1.0	0.00031
IFN-γ/IL-17F	MS^a^ vs HC	0.83	100	58.9	0.23	0.69–0.97	0.00049
IFN-γ/IL-17F	CIS vs MS^a^	0.91	100	75	0.31	0.74–1.0	0.01054
IFN-γ/IL-17F	CIS vs RRMS	0.93	100	75	0.22	0.79–1.0	0.01315
IFN-γ/IL-17F	CIS vs PPMS	0.89	100	75	0.31	0.67–1.1	0.03769
IFN-γ/IL-17F	RRMS vs HC	0.89	100	76.5	0.094	0.77–1.0	0.00065
IFN-γ/IL-17F	PPMS vs HC	0.77	100	58.9	0.23	0.59–0.96	0.03906

*AUC, area under curve; CI, confidence interval; IFN, interferon; CIS, clinical isolated syndrome; RRMS, relapsing–remitting multiple sclerosis; SPMS, secondary progressive multiple sclerosis; PPMS, primary progressive multiple sclerosis; HC, healthy control*.

*^a^MS: pooled RRMS, SPMS, and PPMS patients*.

### Discriminating Value of Th1 and Th17 Lymphocyte Frequencies and the Th1/Th17 Cell Ratio for CIS and MS Phenotypes

We next determined the discriminating value of the Th1 and Th17 lymphocyte frequencies and the Th1/Th17 cell ratio for CIS, MS, and different clinical forms of MS (Table 3). The Th1 cell frequency significantly discriminated RRMS from SPMS (AUC = 0.80) and PPMS (AUC = 0.80). Both the Th1 cell frequency and Th1/Th17 cell ratio significantly differentiated patients with CIS from PPMS (AUC = 0.73 and 0.76, respectively) and SPMS (AUC = 0.82 and 0.75, respectively). The Th1/Th17 cell ratio significantly discriminated CIS from MS (AUC = 0.72). The Th1 cell frequency and Th1/Th17 cell ratio were borderline significant to distinguish CIS from HC and MS (AUC = 0.75 and 0.72, respectively) and CIS from HC (AUC = 0.72), respectively.

**Table 3:** Discriminating value of Th1 and Th17 lymphocyte frequencies and Th1/Th17 cell ratio for MS phenotypes.

Biomarker	Stages	AUC	Sensitivity (%)	Specificity (%)	Cut-off (%)	95% CI	*p*-Value
Th1 cell frequency	CIS vs HC	0.75	86	65	30.47	0.55–0.95	0.0611
	CIS vs MS^a^	0.75	65	77	29.30	0.49–0.81	0.0761
	CIS vs PPMS	0.73	73.3	82.4	26.56	0.54–0.91	0.0299
	CIS vs SPMS	0.82	100	76.5	29.30	0.65–0.99	0.0058
	RRMS vs SPMS	0.80	100	64.7	30.76	0.62–0.96	0.0139
	RRMS vs PPMS	0.80	66.7	88.2	20.14	0.64–0.96	0.0004
Th1/Th17 cell frequency	CIS vs HC	0.72	100	55.6	22.80	0.53–0.92	0.0902
	CIS vs MS^a^	0.72	88.9	55.6	24.96	0.56–0.88	0.0081
	CIS vs PPMS	0.76	75	77.8	12.28	0.59–0.93	0.0107
	CIS vs SPMS	0.75	100	55.6	24.96	0.57–0.93	0.0277

*AUC, area under curve; CI: confidence interval; MS, multiple sclerosis; CIS, clinical isolated syndrome; RRMS, relapsing–remitting multiple sclerosis; SPMS, secondary progressive multiple sclerosis; PPMS, primary progressive multiple sclerosis; HC, healthy control*.

*^a^MS: pooled RRMS, SPMS, and PPMS patients*.

## Discussion

Limited information has been published on the progression of the immune response across the MS clinical course. Very few studies have analyzed the cytokine profile across MS, even fewer have included CIS patients in their analysis, and there are scarce reports analyzing untreated patients. The majority of the studies have compared RRMS and progressive MS (11, 28–30), have analyzed a combination of treated and untreated patients (15, 16, 31), or have analyzed the spontaneous production of cytokines by unstimulated PBMC cultured *in vitro* for 24 h (17). In this cross-sectional study, we found that all untreated patients with CIS and different MS phenotypes exhibited an altered and distinct inflammatory status, but the type of response in these groups differed. These results confirm the notion that MS clinical forms are heterogeneous not only clinically but also immunologically. Furthermore, we provide the first evidence that plasma levels of IFN-γ, the IFN-γ/IL-10 ratio, IL-17F, and the IFN-γ/IL-17F ratio in a subgroup of patients, as well as the frequency of Th1 cells and the Th1/Th17 cell ratio, might represent relevant immunological markers useful for differentiating, monitoring, and potentially predicting the transition to specific MS stages.

We found that IFN-γ levels and the Th1/Th17 cell ratio distinguished CIS patients from MS patients. In addition, IFN-γ levels and the IFN-γ/IL-10 ratio significantly discriminated CIS patients from RRMS patients and this was not due to a difference in the time since last relapse (Table 1). Furthermore, both the frequency of Th1 cells and Th1/Th17 cell ratio differentiated CIS from PPMS. Of significant importance, IFN-γ and the Th1 cell frequency differentiated patients with RRMS from SPMS, a clinical transition for which no immunological biomarkers have been validated yet (14). Notably, the Th1 cell frequency was more accurate than plasma IFN-γ levels. Supporting our data, it has been shown that IFN-γ mRNA levels in unstimulated white blood cells distinguished RRMS from progressive patients (28).

A wide variation in the level of cytokines was found between patients belonging to a same MS subtype, which reflects the heterogeneity of the patient populations. Similarly, Hegen et al. (20) described a high heterogeneity in the cytokine profiles of RRMS patients with levels for some cytokines ranging between very low (less than 50 pg/ml) and very high (higher than 5,000 and 20,000 pg/ml) values as in the case of IL-8 and IL-1RA. As established by these authors, subsets of patients expressing different levels of some cytokines can be associated with different clinical and biological responses to therapy.

Our results show, in a subgroup of patients with IL-17F levels over 250 pg/ml, that this cytokine discriminated CIS from MS, RRMS, PPMS, or HC with high accuracy. Interestingly, Hartung et al. found that patients with levels of IL-17F greater than 200 pg/ml were associated with clinical or radiological disease activity during treatment (32). Together, these results suggest that high levels of IL-17F might represent a biomarker to predict conversion from CIS to MS. Interestingly, in this patient subgroup, the IFN-γ/IL-17F ratio discriminated CIS from MS and RRMS more accurately than IL-17F alone, supporting the notion that in some cases, the ratio between cytokines rather than individual cytokines might better reflect disease progression. Collectively, these results suggest that concomitant analysis of this set of biomarkers might be useful for predicting the clinical evolution of MS. In contrast, some studies have found no significant differences between levels of IFN-γ or IL-17 among different MS phenotypes (15, 16). These contrasting results could be explained by the samples used in the analysis. In those studies, some patients analyzed were treated with DMTs, which could affect the levels of these cytokines. Here, patient samples were collected and analyzed before beginning any treatment and, thus, were not influenced or modified by therapeutic interventions.

In agreement with previous studies (26, 33), we did not find an association between any cytokine levels and clinical parameters for a specific MS phenotype. This might be due to the patient sample size, given that other reports have shown a significant positive correlation between disability and IFN-γ production (34, 35) or between disease activity assessed by MRI and production of either IFN-γ (36) or IL-17 (37).

Whether PPMS is a separate clinical entity has been a long-standing controversy. Clinical, imaging, pathological, and epidemiological data support both possibilities (38). Some authors have proposed that PPMS would be a less inflammatory form of disease (29, 39); whereas other studies have challenged that hypothesis (40, 41). Our findings show that PPMS patients, like other MS phenotypes, have an altered cytokine profile compared to HC showing higher levels of IFN-γ. Similar to the classically inflammatory CIS and RRMS patients, a subgroup of PPMS, and also SPMS, patients presented high levels of IL-17F compared to HC. In contrast, PPMS patients had significantly higher levels of IFN-γ than RRMS and SPMS patients. Furthermore, the IFN-γ/IL-10 ratio was skewed toward a Th1 response in PPMS compared to the other MS phenotypes. Therefore, our findings suggest that PPMS patients exhibited an altered and distinct inflammatory status, differing from other MS phenotypes by their IFN-γ-skewed cytokine profile.

Some reports evaluating the frequency of Th1 cells (29, 30) have not found significant differences among MS subtypes. In contrast, we found a markedly higher Th1 cell frequency in CIS patients than in SPMS and PPMS patients and HC. These conflicting results can be explained by methodological differences. For instance, Duran et al. reported no differences between MS phenotypes for CD3^+^ T cells expressing IFN-γ, whereas we report differences in CD4^+^ Th1 cells (29). Killestein et al. found no significant differences in Th1 frequencies between MS phenotypes; however, this could be explained by their use of a short activation with PMA and ionomycin for 4 h, while we stimulated PBMC for 72 h in the presence of anti-CD3/CD28 (30). It is also worth noting that, in accordance with our results, PPMS patients had a slightly lower Th1 frequency, although the difference did not reach statistical significance (30). We found that CIS patients had a lower Th17 cell frequency than RRMS patients, although this difference was borderline significant (*p* = 0.0496). Another study did not find a significant difference between CIS and RRMS Th17 percentages; however, they studied fewer patients and, unlike our analysis, they separated patients who had experienced a recent relapse from those who had not (42). We found no other differences between MS phenotypes, in accordance with previous findings (43). None of these previous studies examined both Th1 and Th17 cell frequencies in CIS patients. Furthermore, none of them studied PBMC stimulated for 72 h with anti-CD3/CD28. While there may have been some cell death during the 72 h culture, this longer, T lymphocytes specific, and strong stimulus allowed us to analyze greater frequencies of Th1 and Th17 lymphocytes and achieve robust statistical significance. However, lack of viability staining and removal of doublets in our flow cytometry analysis are limitations of our FACS analysis.

Relapsing–remitting multiple sclerosis patients showed a significantly enhanced Th1 cell response compared to SPMS and PPMS, which contradicts the differences observed in IFN-γ levels. This apparent discrepancy can be explained by the production of this cytokine in cells other than Th1, i.e., CD8^+^ T cells and NK cells (27). Interestingly, the Th1/Th17 cell ratio was significantly skewed toward Th1 in CIS compared to RRMS, SPMS, PPMS, and HC.

Together, the evidence described earlier suggests that MS may evolve from a proportionally dominant Th1 to a Th17 response as the clinical course progresses from CIS to RRMS or to PPMS. In turn, the immune response in RRMS patients might polarize toward Th17 as they transition to a secondary progressive disease. Supporting this hypothesis, SPMS and PPMS patients were reported to have significantly decreased levels of IFN-γ mRNA compared to RRMS patients (28), whereas SPMS patients exhibited significantly higher levels of serum IL-17F (44), increased frequency of CD4^+^ROR^+^ T cells (indicative of a Th17 phenotype) (45), and enhanced IL-17-inducible myeloid factors (11), in comparison to RRMS patients. Furthermore, a very recent study reported significantly elevated levels of IFN-γ expression in Vδ1 T cells in recently diagnosed RRMS patients compared to HC. The same group reports that IL-17 and RORγt expressions were low in all T-cell subsets of new MS patients (46). In contrast, Frisullo et al. (17) found that the Th17 response might be more important early in MS while Th1 seemed to be involved both in the early phase and following relapses. However, based on the reported average levels of IFN-γ and IL-17, our estimation of the Th1/Th17 ratio would suggest a slight shift from Th1 to Th17 comparing CIS to RRMS and to SPMS. This shift toward a Th17-mediated immune response as disease naturally evolves to a progressive stage might be, in part, due to Th1 cells being more susceptible to apoptosis than Th17 cells (47–49). However, this shift was not observed in relapsing–remitting or chronic monophasic mouse models (50), although studies measuring the Th1/Th17 ratio remain to be performed in experimental models mimicking the progression between MS phenotypes.

The relatively small sample size of our study is a limitation, and further studies will be needed to validate our findings in larger cohorts. Despite this, we found robust statistical significance with relevant clinical and therapeutic applications. While MRI lesion load, cerebrospinal fluid oligoclonal bands, and IgG index are sensitive tests currently used for MS diagnosis, they lack sensitivity in progressive MS (14, 51) and specificity for differentiating MS from other demyelinating or inflammatory diseases (52). Therefore, the biomarkers found in this study might also be useful for improving differential MS diagnosis. In fact, it has been shown that MS patients have significantly lower serum levels of IFN-γ, IL-10, and IFN-γ/IL-10 ratio than patients with either noninflammatory or inflammatory neurological disorders (53, 54), whereas IL-2 and IFN-γ were reported as good biomarkers in discriminating MS from neuromyelitis optica (NMO) (54). In contrast, recent evidence has shown that failure of therapeutic response to IFN-β in RRMS patients is associated with a Th17 phenotype (20, 25) and that this treatment is ineffective, or can even worsen, other Th17-mediated autoimmune disorders, such as NMO and psoriasis (55). Based on these data, our results might explain, at least in part, why progressive MS patients developing a Th17 disease are nonresponsive to IFN-β and prompt us to evaluate whether the Th1/Th17 cytokine ratio might be used to predict therapy effectiveness.

Overall, our findings contribute to clarify the different roles of the immune system during MS progression, suggesting that the immune response in this disease is a dynamic process that evolves across the clinical course. This novel finding underscores the need to uncover stage-specific immunological pathways that lead to the development of more targeted therapies for patients with different clinical phenotypes.

## Ethics Statement

The study was approved by the Ethics Committee of the Catholic University’s Clinical Hospital, and all patients signed a written informed consent in accordance with the Declaration of Helsinki.

## Author Contributions

Conceived and designed the experiments: RN and CC. Performed experiments: GA, EA, and LIR. Patient recruitment and clinical care: EC, RU, and CC. Analyzed data: GA, PAO, PDS, LV, and RN. Wrote paper: RN and PAO. Critical revision of manuscript: GA, EA, LIR, PDS, LV, EC, RU, and CC.

## Conflict of Interest Statement

CC and EC have had travel expenses reimbursed by Novartis and Biogen, respectively. The other authors declare that they have no competing interests.
