# Supplementary material for: Th1 and Th17 Cells and Associated Cytokines Discriminate among Clinically Isolated Syndrome and Multiple Sclerosis Phenotypes
Source: Front Immunol. 2017 Jun 30;8:753. doi: 10.3389/fimmu.2017.00753 (PMC5491887; doi:10.3389/fimmu.2017.00753)
Supplement: Supplementary file 1 [file image_1.pdf]

frontiers

Supplementary Material

Th1 and Th17 cells and associated cytokines discriminate among clinically isolated syndrome and multiple sclerosis phenotypes

Gabriel Arellano[1], Eric Acuña[1], Lilian I. Reyes[2], Payton A. Ottum[1], Patrizia De Sarno[3], Luis Villarroel[4], Ethel Ciampi[5,6], Reinaldo Uribe-San Martín[5,6], Claudia Cárcamo[5], Rodrigo Naves[1*]

[1]Institute of Biomedical Sciences (ICBM), School of Medicine, Universidad de Chile, Santiago, Chile

[2]Faculty of Science, Universidad San Sebastián, Santiago, Chile

[3]Department of Neurology, University of Alabama at Birmingham, Alabama, USA

[4]Department of Public Health, Pontificia Universidad Católica de Chile, Santiago, Chile.

[5]Department of Neurology, Pontificia Universidad Católica de Chile, Santiago, Chile.

[6]Neurology Service, Hospital Sotero del Río, Santiago, Chile.

*** Correspondence:** Dr. Rodrigo Naves. Immunology Program, Institute of Biomedical Sciences, School of Medicine, Universidad de Chile. Av. Independencia 1027, 8380453, Santiago, Chile. Telephone: 56-2-2978 9603. Fax: 56-2-2978 6979. Email: rodrigonaves@med.uchile.cl

1 Supplementary Figures and Tables

1.1 Supplementary Figures

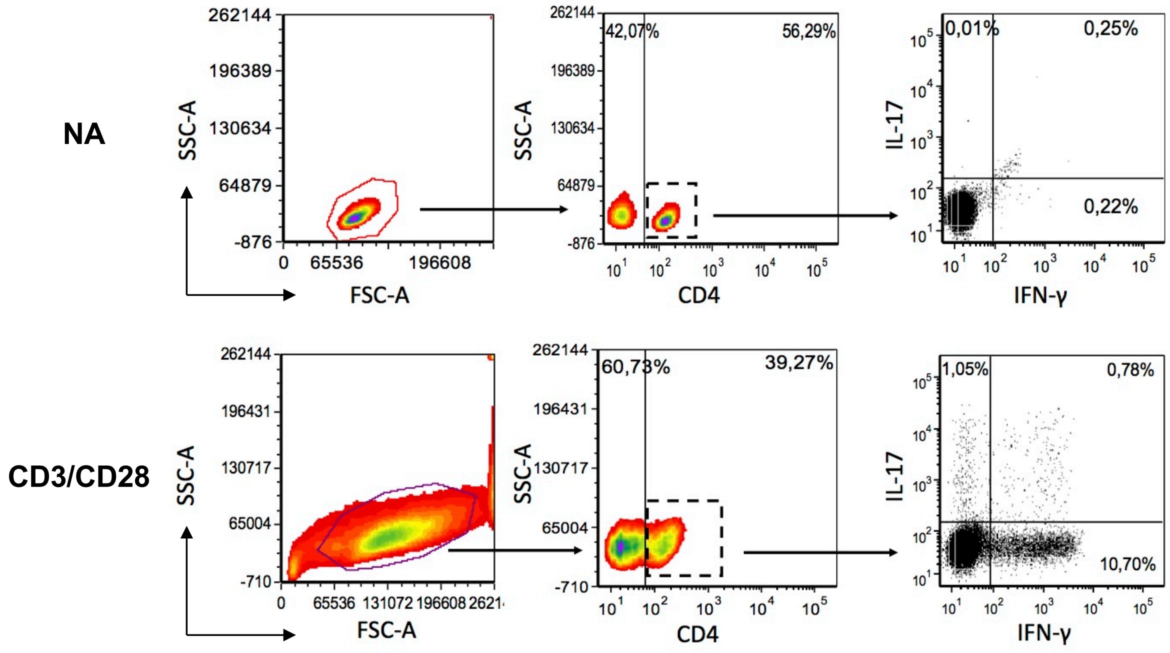

Supplementary Figure 1. Representative flow cytometric analysis of peripheral blood mononuclear cells (PBMC) of a MS patient. Non-activated (NA) or anti-CD3/CD28 mAB (CD3/CD28)-activated cells were cultured for 72 h. Four hours before the completion of PMBC activation, cells were treated with 50 ng/ml Phorbol 12-myristate 13-acetate (PMA), 500 ng/ml Ionomycin and 5 μg/ml Brefeldin A (BFA), or only BFA for the NA control. Then, cells were CD4 cell surface stained and then intracellularly stained for IFN-γ and IL-17A. Lymphocytes were first gated according to forward and side scatter properties and then gated for CD4. The frequency of $CD4^+$ cells producing IFN-γ or IL-17A was finaly determined.
